# Bladder Perforation Secondary to Primary Systemic Amyloidosis

**DOI:** 10.1155/2014/123795

**Published:** 2014-12-22

**Authors:** Christopher J. Dru, Tom S. Feng, Howard H. Kim

**Affiliations:** Department of Urology, Cedars-Sinai Medical Group, Cedars-Sinai Medical Center, 8635 West Third Street, Suite 870, Los Angeles, CA 90048, USA

## Abstract

Amyloidosis is a disorder of protein folding characterized by extracellular aggregation and deposition of amyloid protein fibrils. Light-chain amyloidosis, also known as primary systemic amyloidosis, is the most common form of the disease. We present a case of an 84-year-old male with a history of systemic primary amyloidosis causing genitourinary, cardiac, and autonomic dysfunction who presented with hematuria and hypotension secondary to bladder perforation. He underwent open repair of a large extraperitoneal bladder defect. He ultimately died as a result of medical complications from his disease.

## 1. Introduction

Amyloidosis is the general term used to define a spectrum of protein folding defects characterized by extracellular aggregation of amyloid protein fibrils [1–3]. The pathogeneses of many diseases, with once unknown causes, are now being linked to amyloidosis. At this time, there are over forty diseases associated with the deposition of amyloid plaques [2, 4]. When amyloid fibrils deposit into tissue, the integrity of the organ structure weakens and the ability of the muscle to contract and relax becomes impaired [5]. Amyloidosis can affect any organ in the body and has a wide range of symptomatology. In the genitourinary system, amyloidosis can lead to renal failure and to atonicity and perforation of the bladder [6].  There are only a few case reports of bladder perforation secondary to amyloidosis in the literature. Bladder perforation is generally associated with advanced disease stage and poor prognosis and precipitates death in up to 60% of cases [7, 8].

## 2. Case Presentation

The patient was an 84-year-old male who presented with two days of worsening gross hematuria, dizziness, and fatigue. He had a past medical history significant for systemic amyloidosis with cardiac, renal, genitourinary, gastrointestinal, and autonomic nervous system involvement. The patient's amyloidosis had been in remission for approximately one year with an appropriate response to systemic chemotherapy with lenalidomide (Revlimid), bortezomib (Velcade), and dexamethasone.

Upon the patient's arrival to the hospital, a 22 Fr three-way catheter was placed per urethra with evacuation of several large clots, and continuous bladder irrigation (CBI) was started. His physical examination was unremarkable, but he was found to be anemic (hematocrit 17%) and in renal failure (creatinine 2.3 mg/dL). He was transfused with packed red blood cells but his hematocrit continued to fall. The gross hematuria persisted, and the decision was made to take the patient to the operating room for cystoscopy with clot evacuation and fulguration. An X-ray cystogram was performed that was suspicious for bladder perforation, but no definitive diagnosis could be made given the filling defect from presumed hematoma.

After induction of general anesthesia, a 22 Fr rigid cystoscope was introduced into the bladder. Given the extensive blood clot burden and poor visibility, the 22 Fr scope was exchanged for a 26 Fr rigid cystoscope. Visibility remained poor. An Ellik evacuator and Toomey syringe were used to gently irrigate the bladder. Copious amounts of dark black clots were evacuated, and the output cleared. Again, the rigid cystoscope was introduced with visualization of diffuse trabeculations and several diverticula. At the dome of the bladder, there was a sizeable circular defect filled with clot and fibrinous material. The cystoscope was removed, and a Foley catheter was inserted. Approximately 300 mL of dilute contrast was instilled into the bladder, and a cystogram was obtained with the fluoroscopic unit. The cystogram demonstrated a minimal amount of perivesical contrast extravasation. Given continued bleeding and the size of the perforation, the decision was made to transfer the patient to the main operating room for exploratory laparotomy, control of bleeding, and repair of bladder defect.

An 8 cm midline infraumbilical incision was made, and dissection was carried down through the rectus fascia. The space of Retzius was entered with immediate evacuation of fluid and blood clot. A 4 cm extraperitoneal defect at the dome of the bladder was noted. The perforation appeared to be nonacute in nature, as there was organized clot and chronic tissue inflammation. The only site of active bleeding was on the edge of the bladder defect. Hemostasis was achieved with electrocautery. The bladder defect was closed in a watertight fashion in three layers with absorbable suture.

A 24 Fr three-way catheter was placed per urethra and was used to irrigate the bladder to demonstrate resolution of bleeding and confirm a watertight bladder closure. A 19 Fr Blake drain was placed in a dependent position adjacent to the bladder repair through a separate stab incision and was secured to the skin. The rectus muscle, fascia, and skin were closed in the usual fashion. At the end of the case, the patient became acutely hypotensive. A central line was placed and intravenous vasopressors were started. He was transferred to the surgical intensive care unit for hemodynamic and respiratory monitoring.

On postoperative day 1, his hemodynamics improved, and he was extubated. His urine was clear on minimal CBI. He was alert and talkative and in good spirits. However, over the course of several days, his clinical condition worsened. He developed massive hematemesis requiring esophagogastroduodenoscopy that demonstrated diffuse gastric bleeding. He was reintubated for respiratory distress and required higher doses of vasopressor support for hypotension. His renal function deteriorated. His hematocrit continued to drop despite aggressive blood transfusions. After a discussion of his poor prognosis with his family, the decision was made to change his code status to DNAR and he was palliatively extubated. He died several hours later, comfortably, with his family.

## 3. Discussion

Amyloidosis is a devastating disease with a wide range of subtypes and clinical manifestations. As of 2010, there were 27 unique human amyloid protein structures stemming from 18 specific amyloid protein gene mutations [9]. Our patient suffered from light-chain amyloidosis, also known as primary systemic amyloidosis, the most common form of the disease [10]. This subtype is characterized by the production of monoclonal light chains by a small focus of abnormal plasma cells in the bone marrow with subsequent deposition in the vital organs [11, 12]. Deposition and accumulation of these proteins lead to organ dysfunction, particularly from cardiac and renal involvement [6, 13]. Diagnosis is made by tissue biopsy followed by Congo red stain with the characteristic apple green birefringence under polarized light [14]. The mainstay of treatment for AL amyloidosis is chemotherapy with a combination of dexamethasone and either bortezomib or lenalidomide [15]. While treatment has been effective achieving durable complete remission, the organ damage is often irreversible and fatal. In many cases, the diagnosis is made late, and supportive care is the only option for the patient. In our patient, he had a history of biopsy-proven amyloidosis in the bladder, kidneys, colon, heart, and nervous system, all consistent with a diagnosis of advanced systemic amyloidosis.

The management of bladder perforation depends upon the location and nature of the defect. There are no specific guidelines in the context of amyloidosis; however, the clinician must be aware that this patient population will have delayed and often poor wound healing [16]. In general, an uncomplicated extraperitoneal bladder perforation can be managed with urinary catheter drainage for 10–14 days followed by a cystogram to confirm resolution of contrast extravasation [17, 18]. Complicated extraperitoneal and all intraperitoneal bladder perforations are typically managed by open surgical repair of the bladder with a watertight closure in three layers with absorbable suture [19, 20]. Our patient had a complicated extraperitoneal bladder defect requiring open surgical repair given his systemic amyloidosis, hemodynamic instability, and active bleeding. His death was precipitated by the continued and prolonged stress on his already damaged heart in conjunction with multiorgan failure as a result of the acute blood loss, autonomic dysfunction, and inability to maintain adequate mean arterial blood pressure and tissue perfusion.

Research is ongoing in the field of amyloidosis, and much progress is being made in therapeutics. Recent studies have hypothesized and demonstrated that the misfolding of proteins may be a reflection of the cellular microenvironment as opposed to a malignant pathologic process or specific gene mutation as once previously thought [3, 21, 22]. It is of paramount importance that healthcare providers of diverse specialties including urology familiarize themselves with the diagnosis and management of amyloidosis given the varied morbidity, and often mortality, of this evolving disease.
